# C-Myc-dependent repression of two oncogenic miRNA clusters contributes to triptolide-induced cell death in hepatocellular carcinoma cells

**DOI:** 10.1186/s13046-018-0698-2

**Published:** 2018-03-09

**Authors:** Shu-Guang Li, Qian-Wei Shi, Ling-yan Yuan, Li-ping Qin, Yan Wang, Yu-Qing Miao, Zhe Chen, Chang-Quan Ling, Wen-xing Qin

**Affiliations:** 1 Department of Traditional Chinese Medicine, Changhai Hospital, Second Military Medical University, Shanghai, People’s Republic of China; 2 Department of Endocrinology, General Hospital of Wuhan, People’s Liberation Army, Wuhan, People’s Republic of China; 3 Guang An’ men Hospital, China Academy of Chinese Medical Sciences, Beijing, People’s Republic of China; 4 Department of oncology, Changzheng Hospital, Second Military Medical University, Shanghai, People’s Republic of China

**Keywords:** Liver cancer, Triptolide, miRNA cluster, Apoptosis

## Abstract

**Background:**

Triptolide is a structurally unique diterpene triepoxide with potent antitumor activity. However,the effect and mechanism of triptolide on hepatocellular carcinoma (HCC) is not well studied.

**Methods:**

Cells were treated with triptolide, and the anti-HCC activity of triptolide was evaluated using flow cytometry, western blot, and xenograft studies. MicroRNA microarray and quantitative reverse-transcription polymerase chain reaction was used to identify differential microRNAs induced by triptolide. Chromatin immunoprecipitation assay was employed to study the interaction between c-Myc and genomic regions of miR106b-25. MicroRNAs overexpression and knockdown experiments were performed to determine the role of these microRNAs in triptolide-induced apoptosis.

**Results:**

Triptolide inhibited cell proliferation and induced marked apoptosis in multiple HCC cell lines with different p53 status. Several signaling molecules involved in different pathways were altered after the treatment of triptolide. Xenograft tumor volume was significantly reduced in triptolide-treated group compared with vehicle control group. Two miRNA clusters, miR-17-92 and miR-106b-25, were significantly suppressed by triptolide, which resulted in the upregulation of their common target genes, including BIM, PTEN, and p21. In HCC samples, high levels of these miRNA clusters correlated with shorter recurrence free survival. Triptolide inhibited the expression of theses miRNAs in a c-Myc-dependent manner, which enhanced triptolide-induced cell death. We further showed that triptolide down-regulated the expression of c-Myc through targeting ERCC3, a newly identified triptolide-binding protein.

**Conclusions:**

The triptolide-induced modulation of c-Myc/miRNA clusters/target genes axis enhances its potent antitumor activity, which indicates that triptolide serves as an attractive chemotherapeutic agent against HCC.

**Electronic supplementary material:**

The online version of this article (10.1186/s13046-018-0698-2) contains supplementary material, which is available to authorized users.

## Background

Hepatocellular carcinoma (HCC) is the third leading cause of cancer death worldwide with annual death exceeding 600,000 [1]. HCC is associated with very poor prognoses because of its aggressive growth, metastasis, and resistance to most current therapeutic approaches [2]. Therefore, there is an urgent need to develop effective therapeutic strategies for the large number of HCC patients [3].

MicroRNAs (miRNAs) are endogenous ∼23 nt RNAs that negatively regulate gene expressions by pairing to the mRNAs of protein-coding genes to direct their posttranscriptional modifications [4, 5]. As key negative regulators in gene expression, miRNAs play an important role in many cellular processes, such as differentiation, proliferation, and apoptosis [5]. Importantly, a large body of evidence has shown that miRNAs regulate molecular pathways in cancer by targeting various oncogenes and tumor suppressors, which play a vital role in cancer development and progression [6]. Not surprisingly, miRNAs have also been discovered to be aberrantly expressed in HCC and some of them are functionally implicated in hepatocarcinogenesis and progression [7]. Combinations of genomic analyses and functional studies have identified some miRNAs, such as miR-17-92, miR-21, and miR-221, which function as oncogenes in initiation and maintenance of HCC [7, 8]. Meanwhile, some miRNAs, including let-7, miR-122 and miR-26, have been identified as tumor suppressors [9]. Therefore, targeting oncogenic miRNAs and restoring tumor-suppressive miRNAs would be a reasonable therapeutic strategy for HCC patients.

Triptolide is a structurally unique diterpene triepoxide isolated from Tripterygium wilfordii Hook F, a Chinese medicinal plant used for treating a wide range of diseases for centuries [10]. Triptolide has been shown to possess potent anti-inflammatory, immunosuppressive and anticancer activity [11]. The antitumor activity range is quite broad in that triptolide is capable of killing cancer cells which originate from various tissues, including blood, colon, breast, brain, ovary, kidney and prostate, with IC_50_ values in nanomolar range [12]. The mechanisms of antitumor activity of triptolide have been extensively investigated in the past few decades. Triptolide is demonstrated to inhibit transcription, which was initially attributed to affectingspecific transcription factors. But recently it was revealed to lead to global transcription inhibition via targeting RNA polymerase I and II [13]. More recently, Titov et al. reported that triptolide covalently bound to a human 90 kD protein ERCC3 (also known as XPB) and inhibited its DNA-dependent ATPase activity, which led to the inhibition of RNA polymerase II–mediated transcription [14].

It seems that the transcription inhibition accounts for most of the aforementioned biological activities of triptolide. Besides down-regulating the expression of numerous genes, triptolide has also been revealed to increase the mRNA or protein levels of various molecules, including p53, RIZ1 and HIF-1a, which contributes to the anticancer activity [15–17].

To explain this discrepancy, we hypothesized that triptolide inhibited the transcription of some oncogenic miRNAs, which in turn increased the protein level of their target genes. We used miRNA microarrays and found that the miRNAs expression profiles were significantly altered by the treatment of triptolide. Among the modulated miRNAs, up to 94% are down-regulated. Two oncogenic miRNA cluster, mir-17-92 and mir-106b-25, were down-regulated by triptolide simultaneously. On the contrary, the putative target genes of these miRNA clusters were up-regulated by triptolide. We further proved that both of the two miRNA clusters were directly transactivated by c-Myc, and triptolide down-regulated the expression of these miRNA clusters in a c-Myc dependent manner. Importantly, the modulation of c-Myc/miRNA clusters /target genes cascade contributed to triptolide-induced cell death.

## Methods

### Small interfering RNA (siRNA) and antisense oligonucleotides (ASOS) synthesis

The siRNAs specifically targeting *MYC*, *E2F1*, *ERCC3* and control siRNA (the sequences were depicted in Additional file 1: Table S2) and the antisense oligonucleotides for miRNAs were synthesized by GenePharma (Shanghai, China).

### Construction of vectors

The complementary DNA encoding ERCC3 and c-Myc was PCR-amplified by the Pfu Ultra II Fusion HS DNA Polymerase (Agilent Technologies, Palo Alto, CA), and was subcloned into the pcDNA3.1 vector (Invitrogen, Carlsbad, CA). The miR-17-92 and miR-106b-25 cluster were amplified from genomic DNA and cloned into pcDNA3.1 (Invitrogen, Carlsbad, CA). The promoter region of the *MCM7*, from − 68 to + 115 bp upstream of the transcription start site, was amplified and subcloned into the pGL3 basic firefly luciferase reporter (Promega, Madison, WI). pGL3 basic vector was used as a negative control. The pGL3 construct containing the MCM7 promoter with point mutations in the c-Myc binding sites were synthesized using a QuikChange Site-Directed Mutagenesis kit (Stratagene, La Jolla, CA). The two newly constructed reporter plasmids were designated as pGL3-WT and pGL3-MUT, respectively. All of the primer sequences were presented in Additional file 1: Table S2.

### Cell culture and transfection

Immortalized normal human liver cell lines L02 and Liver cancer cell lines SMMC-7721, MHCC-97H, and LM3 were obtained from Cell Bank of Type Culture Collection of the Chinese Academy of Sciences (Shanghai Institute of Cell Biology, Chinese Academy of Sciences). HepG2 and Hep3B were purchased from American Type Culture Collection (Manassas, VA). The cells were cultured in Dulbecco’s Modified Eagle Medium (Gibco BRL) with 10% fetal bovine serum (Gibco BRL) and maintained in an atmosphere of 5% CO_2_ in a humidified 37 °C incubator. Transfections were performed using the Lipofectamine 2000 kit (Invitrogen, Carlsbad, CA) according to the manufacturer’s instructions.

### Determination of cell proliferation

Cell proliferation was determined using the Cell Counting Kit-8 (Dojindo, Kumamoto, Japan). 1 × 10^4^ cells per well were seeded into a 96-well plate and allowed to adhere overnight. After treatment with triptolide at various concentrations for 24 and 48 h, 10 μL of CCK-8 solution was added to each well of the plate. Plates were incubated at 37 °C for 1 h, after which the absorbance at 450 nm was measured using a microplate reader (Biotek, Winooski, VT). All experiments were performed in quadruplicate and repeated at least three times.

### Cell death assay

Cells were stained with fluorescein isothiocyanate (FITC)-conjugated antiannexin V antibody, which was labeled in combination with propidium iodide (PI) according to the manufacturer’s instructions (KeyGEN, Nanjing, China), then analyzed by FACS (BD, San Diego, CA). Cell death percentage corresponded to Annexin-V (+)/PI (+) cells. Apoptotic cell death percentage represents Annexin-V (+)/PI (−)-stained cells.

### Cell cycle analysis

Cells (5 × 10^6^ cells/well) were treated with indicated concentrations of triptolide for 24 h and were harvested. After being washed, the cells were fixed with 70% ice-cold ethanol and maintained overnight at 4 °C. These cells were collected and resuspended in phosphate buffered saline containing 40 μg/mL PI, 0.1 mg/mL RNase and were incubated at 37 °C for 30 min. The cells were evaluated by BD FACSCalibur (San Diego, CA). Data were collected from at least 10,000 cells for each sample. The cell cycle distribution was analyzed using CELLQuest Software.

### Chromatin immunoprecipitation assay

Chromatin immunoprecipitation (ChIP) assays were performed according to the manufacturer’s instructions (Millipore, USA). ChIP-derived DNA was quantified using RT-PCR (Applied Biosystems). The primers specific for the *MCM7* promoter or the *ERCC3* promoter were listed in Additional file 1: Table S2.

### qRT-PCR

Total RNA from different cell lines and human tissues were extracted using Trizol reagent (Invitrogen, Carlsbad, CA). qRT-PCR was performed using an ABI 7300 Fast Real-Time PCR System (Applied Biosystems, Foster City, CA) and SYBR Green PCR kit (Takara, Otsu Shiga, Japan). The gene-specific stem–loop reverse transcriptase (RT) primers for miRNA were purchased from RiboBio (Guangzhou, China). The primer sequences for mRNA were provided in Additional file 1: Table S2.

### Protein extraction and western blot analysis

Total cell lysates were prepared in 1× sodium dodecyl sulfate buffer. Identical quantities of proteins were separated by sodium dodecyl sulfate-polyacrylamide gel electrophoresis and transferred onto polyvinylidene fluoride membranes. After being blocked, the membrane was incubated with specific primary antibodies overnight, washed, incubated with horseradish peroxidase-conjugated secondary antibody, and detected with enhanced chemiluminescence solution (Thermo Scientific, Rockford, IL).

### Generation of luciferase-expressing cell line HepG2-luc

Recombinant lentiviruses containing the firefly luciferase gene were purchased from GeneChem (Shanghai, China). To generate the stable cell line, 4 × 10^5^ HepG2 cells were transfected with 2 × 10^6^ transducing units of lentiviruses and were selected with 2 μg/ml puromycin for two weeks. Isolated clones were screened for their luciferase activities using an IVIS Spectrum (Caliper Life Sciences, MA).

### Luciferase reporter assay

C-Myc transcriptional activity was assessed using a dual luciferase reporter assay system. Briefly, pMyc-TA-luc (Beyotime, Nantong, China) and pRL-TK plasmids were cotransfected into cultured cells by Lipofectamine-mediated gene transfer. Then the transfected cells were treated with various concentrations of triptolide. To evaluate the transcription activity of these reporter plasmids that carried wild type or mutant MCM promoter region, pGL3-WT or pGL3-MUT, along with pRL-TK were cotransfected into pcDNA-c-Myc or pcDNA-Mock-transfected cells. Luciferase assays were performed with the dual luciferase reporter assay system (Promega, Madison, WI). The relative luciferase activity was normalized with renilla luciferase activity.

### miRNA expression profiling

HepG2 cells (5 × 10^6^ cells /well) were seeded into a 6-well plate. After incubation for 12 h, the cells were exposed to various concentrations of triptolide (100 nM, 200 nM) for 12 and 24 h. DMSO treatment served as a negative control. Total RNA were isolated with the Trizol reagent (Invitrogen). MicroRNA microarray analysis was performed using the miRCURY LNA Array (Exiqon, Vedbaek, Denmark). The RVM f-test was applied to determine the differentially expressed genes. After signals of low intensity were filtered out, the differentially expressed genes were selected according to the *P*-value and fold-change threshold (≥2).

### Xenograft studies

BALB/c nude mice, aged 4–5 weeks, were obtained from Shanghai Silaike Animal Experimental Center. All animal experiments were performed in accordance with the guidelines of the Second Military Medical University Animal Care Facility and the National Institutes of Health. To establish a xenograft model, 1 × 10^7^ HepG2-luc cells per mouse were injected subcutaneously into the right flanks of nude mice. After growing for 7 days, mice with visible tumor xenografts were randomly divided into three treatment groups: (a) negative control treated with DMSO, (b) positive control treated with cisplatin and (c) triptolide-treated mice. Mice were given daily i.p. injections of triptolide (0.2 mg/kg), vehicle (DMSO) or cisplatin (1 mg/kg) for 14 days, respectively. Tumor size was assessed every 2 days using a digital caliper; the volume = (length × width^2^)/2. The in vivo imaging of tumors was performed using IVIS Imaging Systems (Alameda, CA). Implanted tumors were rapidly excised into two fragments. One fragment was stored in RNAlater for RNA extraction and analysis of miRNA by real-time PCR. The other was fixed in formalin and embedded in paraffin for subsequent TUNEL or immunohistochemistry staining.

### TUNEL assay

Paraffin-embedded tumor tissue sections from control and triptolide-treated mice were processed for the terminal deoxynucleotidyl transferase–mediated dUTP nick end labeling (TUNEL) assay according to the manufacturer’s instructions (Chemicon, Temecula, CA).

### Immunohistochemistry

The xenograft tumor slides were incubated with specific antibodies. Anti-rabbit or anti-mouse peroxidase-conjugated secondary antibodies (Cell Signaling Technology, Beverly, MA) were applied. Diaminobenzidine colorimetric reagent solution from Sigma (St Louis, MO) was used. Slides were counterstained with hematoxylin (Sigma Chemical Co). Staining of the whole tissue sections was examined and scored by 2 independent observers.

### Clinical samples

Tumor tissues and paired adjacent non-tumor tissues from 30 HCC patients were obtained during surgery. Patient clinical details were summarized in Additional file 2: Table S1. All patients underwent resection of the primary tumor at Changhai Hospital between 2009 and 2011. Patient samples were obtained following informed consent according to an established protocol approved by the Ethic Committee of Changhai Hospital.

### Statistical analysis

The statistical differences between the groups were analyzed by student *t* test or one-way analysis of variance. Kaplan–Meier analysis was used to determine survival. Log-rank test was used to compare patients’ survival between subgroups. The statistical correlation between the clinical parameters of HCC and the miRNAs expression levels in tissue sections was analyzed by the chi-square test. All *P* values were obtained using the SPSS 16.0 software package (SPSS, Chicago, IL). *P* < 0.05 was considered statistically significant.

## Results

### The anti-HCC effect of triptolide in vitro and in vivo

**Fig. 1:**
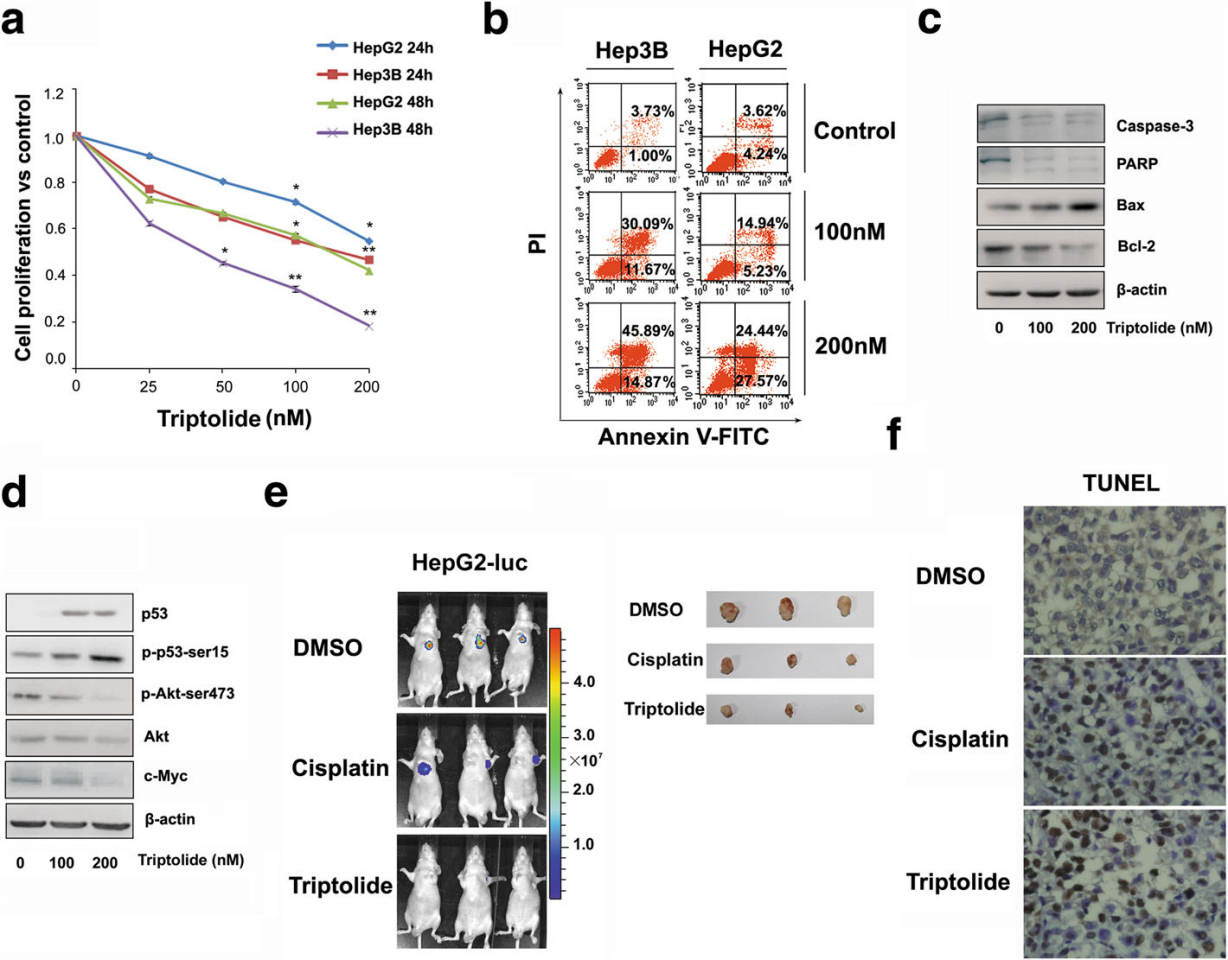
Triptolide showed potent anti-HCC activity both in vitro and in vivo. **a** Treatment of HepG2 and Hep3B cells with triptolide for 24 and 48 h showed a significant decrease in cell proliferation. The bars represent mean ± standard error of the mean, *n* ≥ 3. P*<.05, **P<.01 (t test). **b** Flow cytometric analysis revealed that the treatment with triptolide for 48 h significantly increased Annexin V staining in HepG2 and Hep3B cells. **c** and **d** Western blot showed the treatment of triptolide for 24 h induced the activation of some apoptotic markers and altered the expression and phosphorylation of some key regulators of apoptosis and cell growth in HepG2 and Hep3B cells. **e** The in vivo imaging of implanted tumors was performed to monitor the growth of xenografts. Representative imaging (left panel) and the photographs of corresponding tumors dissected from nude mice (right panel) indicated that triptolide significantly reduced tumor volume. **f** Representative TUNEL assays showed a significant induction of apoptosis in triptolide-treated group compared with DMSO-treated group (original magnification 400×)

We first examined the effect of triptolide on the proliferation of two HCC cell lines. HepG2 (wild-type p53) and Hep3B (deleted p53) cells were incubated in medium containing triptolide at concentrations of 25 to 200 nM for 24 and 48 h. As shown in Fig. 1a, the intervention of triptolide significantly inhibited both HepG2 and Hep3B cell proliferation in a dose- and time-dependent manner. The effect of triptolide on other commonly used HCC cell lines, such as Huh7, SMMC-7721, LM3, was also examined, and similar results were observed (data not shown). Furthermore, considering the different p53 status of HepG2 and Hep3B cells, we concluded that triptolide induced cell proliferation inhibition of HCC cells in a p53- independent manner.

Next, triptolide-induced apoptosis was measured with various methods. In Fig. 1b, the induction of apoptosis was measured by flow cytometry and quantitatively expressed as the percentage of cells with positive Annexin-V. After 48 h, triptolide dose-dependently increased Annexin-V in both HepG2 and Hep3B cells, demonstrating that it induced cell apoptosis. Moreover, the treatment of triptolide showed a substantial increase of cleaved caspase-3, caspase-9 and PARP, which indicated that these cells underwent apoptosis. Simultaneously, some pro-apoptotic regulators, such as Bax, 0 and p53, were up-regulated by triptolide. Conversely, the expression of some anti-apoptotic proteins, including bcl-2, Akt, and c-Myc, were significantly diminished (Fig. 1c and d).

Considering the up-regulation of cell cycle regulator p21 was also observed (Additional file 3: Figure S1C), we next evaluated the effect of triptolide on the cell cycle distribution. It was reported that triptolide induced cell cycle arrest in HepG2 cells at relatively lower concentrations. Inconsistent with this study [18, 19], the cell cycle distribution in liver cancer cells was not significantly altered in the presence of triptolide (Additional file 3: Figure S1A and B). In addition, the expression of other key cell cycle regulators, such as cyclin A, cyclinD, and Rb showed no significant alterations after incubation with triptolide (Additional file 3: Figure S1C). We therefore concluded that triptolide did not induce cell cycle arrest at relatively higher concentrations.

To determine the in vivo effect of triptolide on HCC cells, HepG2-luc cells (HepG2 cells stably transfected with luciferase) were injected subcutaneously into the right flanks of nude mice, as described in Methods. Mice were then randomized into three groups treated with DMSO, cisplatin and triptolide, respectively. After a daily injection of triptolide (0.2 mg/kg) for 14 days, the luciferase activity and tumor volume was significantly reduced (Fig. 1e), as compared with controls injected with DMSO. Surprisingly, triptolide even exhibited stronger anticancer activity than that of cisplatin, a widely used chemotherapy agent. Importantly, the treatment of tripotide exhibited no evidence of altered liver and kidney function in nude mice (Additional file 4: Figure S2). Taken together, these findings indicated that triptolide exhibited potent anti-HCC activity both in vitro and in vivo.

### Triptolide inhibited the transcription of miR-17-92 and miR-106b-25

**Fig. 2:**
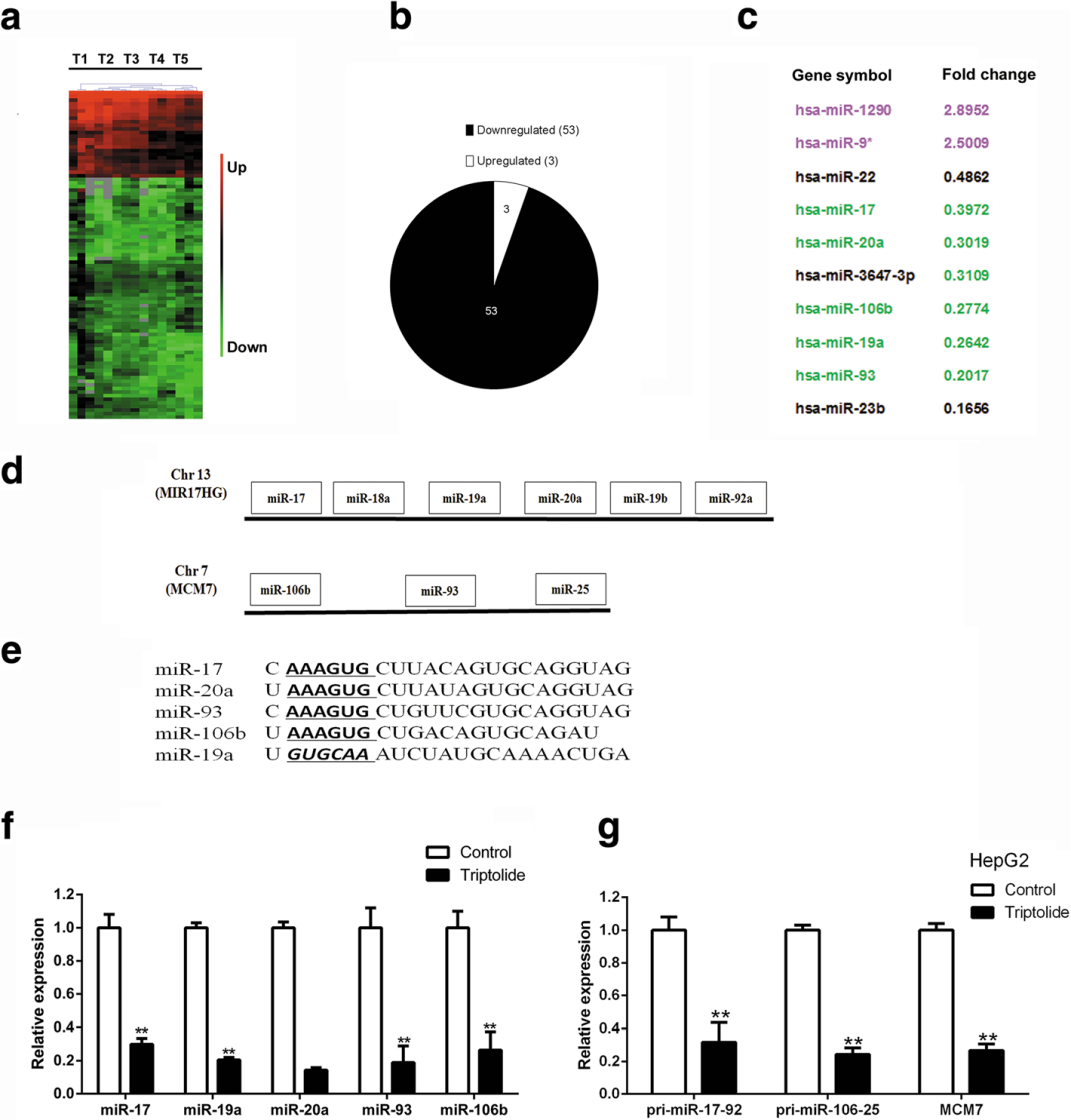
Triptolide inhibited the expression of miR-17-92 and miR-106b-25. **a** Representative heat map of microarray analyses of triptolide-treated HepG2 cells. Five groups of HepG2 cells were treated as follows: T1 (DMSO treated), T2 (100 nM for 12 h), T3 (100 nM for 24 h), T4(200 nM for 12 h), T5 (200 nM for 24 h). Each group contained three biological replicates. **b** The pie chart represented the proportion of all differentially expressed miRNAs induced by triptolide. **c** The top ten high intensity differentially expressed miRNAs induced by triptolide. **d** The genomic organization and primary transcript structures of human mir-17-92 and miR-106b-25. qRT-PCR was performed to validate the profile data. **e** The seed sequence of five differentially expressed miRNAs. **f**–**g** Triptolide significantly down-regulated the expression of indicated mature miRNAs (left panel) and primary transcripts (right panel). The bars represented mean ± standard error of the mean, n ≥ 3. P*<.05, **P<.01 (t test)

It was reported that triptolide-induced transcription inhibition accounted for most of its biological activities [11]. However, besides down-regulating the expression of numerous genes, triptolide increased the mRNA or protein levels of several molecules, and such an increase contributed to the anticancer activity of triptolide [11]. Therefore, we hypothesized that triptolide could also inhibit the expression of specific negative regulators, such as miRNAs, which in turn up-regulate their target genes and subsequent biological effects. To test this hypothesis, we performed miRNA microarray to identify triptolide-regulated miRNAs in HepG2 cells. The clusters analysis revealed that the treatment of triptolide induced significant changes in miRNA expression (Fig. 2a), especially when cells were exposed to triptolide at a concentration of 200 nM for 24 h. We identified 56 differentially expressed miRNAs, among which 53 miRNAs were down-regulated (Fig. 2b). We ranked the top ten differential miRNAs according to their raw signal intensity (Table 1), and found that five miRNAs, including miR-17, miR-19, miR-20, miR-93, and miR-106b belonged to two oncogenic miRNA clusters, miRNA-17-92 and miRNA-106b-25 (Fig. 2c and d). Among these miRNAs, four miRNAs shared same seed sequence (Fig. 2e). To first validate the profile data, we performed stem loop quantitative RT-PCR (qRT-PCR) analysis in cells treated with triptolide. As shown in Fig. 2f, the expression of five miRNAs above mentioned was significantly repressed by triptolide.

**Table 1:** Top Ten High Intensity Differentially Expressed miRNAs

	Fold change	FDR	*P*-value
hsa-miR-1290	2.8952	0.0981	0.0035
hsa-miR-1246	1.6527	0.1122	0.0050
hsa-miR-22	0.4862	0.1434	0.0116
hsa-miR-17	0.3972	0.1036	0.0041
hsa-miR-20a	0.3019	0.2280	0.0407
hsa-miR-106b	0.2774	0.1395	0.0106
hsa-miR-19a	0.2642	0.1036	0.0042
hsa-miR-93	0.2017	0.2176	0.0348
hsa-miR-23b	0.1656	0.2272	0.0402
hsa-miR-16	0.1429	0.1625	0.0152

The miR-17-92 cluster, including miR-17, miR-18a, miR-19a, miR-20a, miR-19b, and miR-92a, is encoded by the Chromosome 13 open reading frame 25,and the miR-106b-25 cluster is composed of the highly conserved miR-106b, miR-93, and miR-25 (Fig. 2d). Thus, the effect of triptolide on the remaining members of miR-17-92 and miR-106b-25 was also examined, and similar observations were obtained (data not shown). However, because of the lower raw signal intensity, these members were excluded from the list of differentially expressed miRNAs. Since posttranscriptional cleavage is required for the maturation of miRNA, we speculated that the posttranscriptional modulation also played a major role in determining the levels of mature miRNAs in triptolide-treated cells. To determine whether triptolide down-regulates the expression of these miRNAs through inhibiting the transcription of their primary transcripts, we performed qRT-PCR to quantify the primary transcripts of two miRNA clusters. As expected, the transcription of primary transcripts was significantly inhibited by triptolide (Fig. 2f, right panel). Furthermore, the expression of pri-miR-17-92 and pri-miR-106b-25 was also down-regulated in triptolide-treated xenografts when compared with DMSO-treated tumor samples (Additional file 5: Figure S3). Together, triptolide down-regulated the expression of five miRNAs belonging to miR-17-92 and miR-106b-25 both in vitro and in vivo.

### Down-regulation of miRNA cluster enhanced the anti-HCC activity of triptolide

**Fig. 3:**
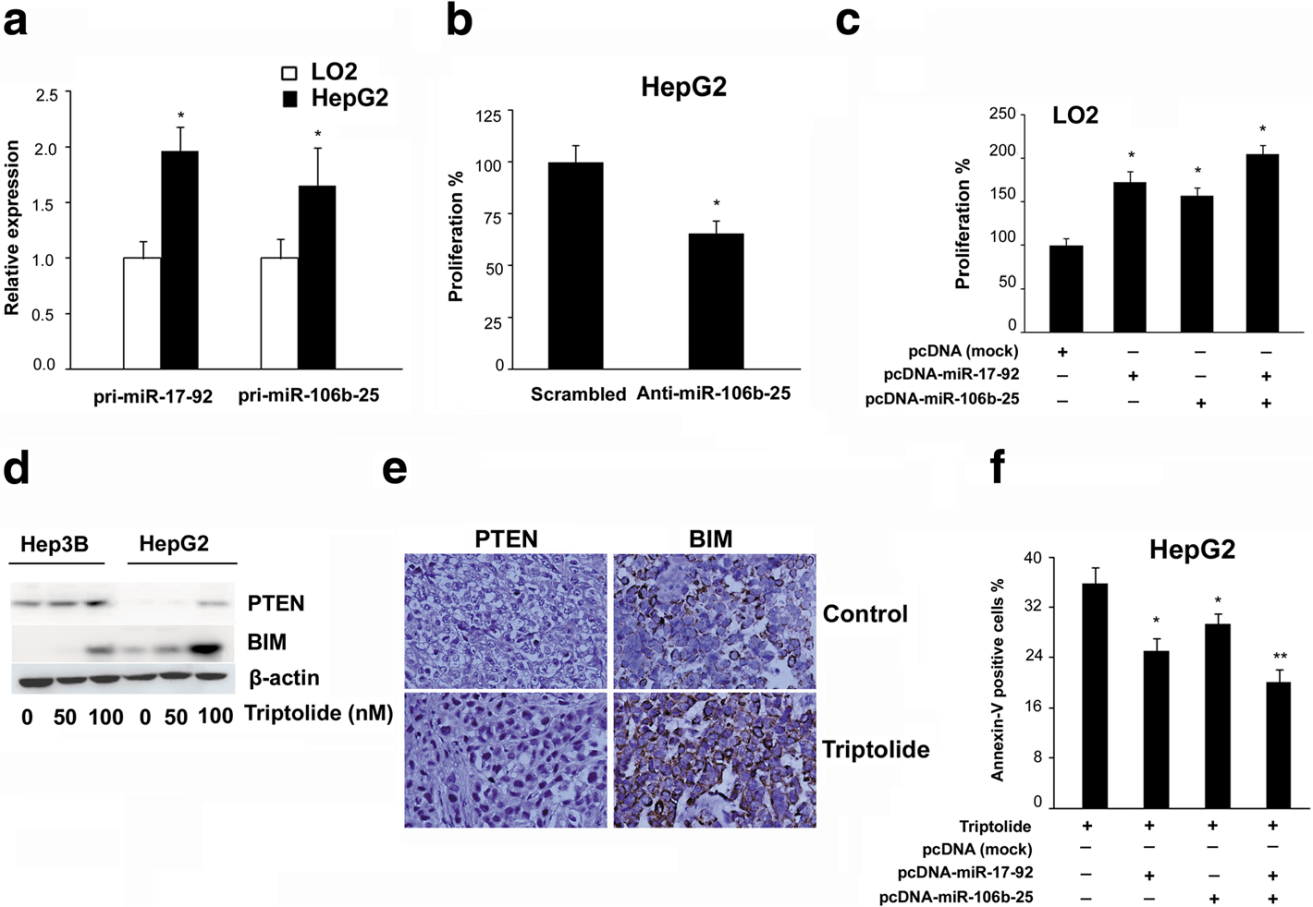
Down-regulation of miR-17-92 and miR-106b-25 contributed to antitumor activity of triptolide in HepG2 cells. **a** qRT-PCR was performed to compare the expression of pri-miR-17-92 and pri- miR-106b-25 in LO2 and HepG2 cells. **b** Knockdown of miR-106b-25 using antisense oligonucleotides reduced HepG2 cell proliferation. **c** Ectopic expression of miR-17-92 and miR-106b-25 with corresponding expression vector enhanced the proliferation of LO2 cells. **d** and **e** Triptolide up-regulated the expression of PTEN and BIM in HepG2 cells and implanted tumors. **f** Forced expression of miR-17-92, miR-106b-25, or the combination of miR-17-92 and miR-106b-25 protected HepG2 cells from triptolide-induced apoptosis. The bars represented mean ± standard error of the mean, n ≥ 3. *P<.05, **P<.01 (t test)

Next, we were interested in the biological effect of downregulation of these miRNAs. A large number of studies have shown that miR-17-92 and its paralog miR-106b-25 played an important role in tumorigenesis. The overexpression of these miRNAs has been observed in diverse tumor subtypes. Consistent with previous observations that these miRNAs were evaluated in HCC cells [20], the miR-17-92 and miR-106b-25 expression in HepG2 cells was significantly higher than that in immortalized normal liver LO2 cells (Fig. 3a). It was reported that knockdown of miR-17-92 reduced the proliferation of HepG2 cells. We demonstrated that knockdown of miR-106b-25 with antisense oligonucleotides also decreased the proliferation of HepG2 cells (Fig. 3b). Conversely, overexpression of miR-17-92 or (and) miR-106b-25 promoted the proliferation of LO2 cells (Fig. 3c), suggesting that these miRNAs could be therapeutic targets for HCC. As shown in Fig. 3d and e, triptolide down-regulated miR-17-92 and miR-106b-25 expression simultaneously, which resulted in an increase of their common target genes, including BIM and PTEN, both of which are well known pro-apoptotic proteins. Furthermore, the overexpression of these miRNAs using two mammalian expression vectors, pcDNA-miR-17-92 and pcDNA-miR-106b-25, protected HepG2 cells from triptolide-induced apoptosis, thus demonstrating that the inhibition of these miRNA clusters promoted triptolide-induced apoptosis. In conclusion, down-regulation of two oncogenic miRNA clusters enhanced the anti-HCC activity of triptolide (Fig. 3f).

### Triptolide inhibited the transcription of miR-17-92 and miR-106b-25 through repressing c-Myc

**Fig. 4:**
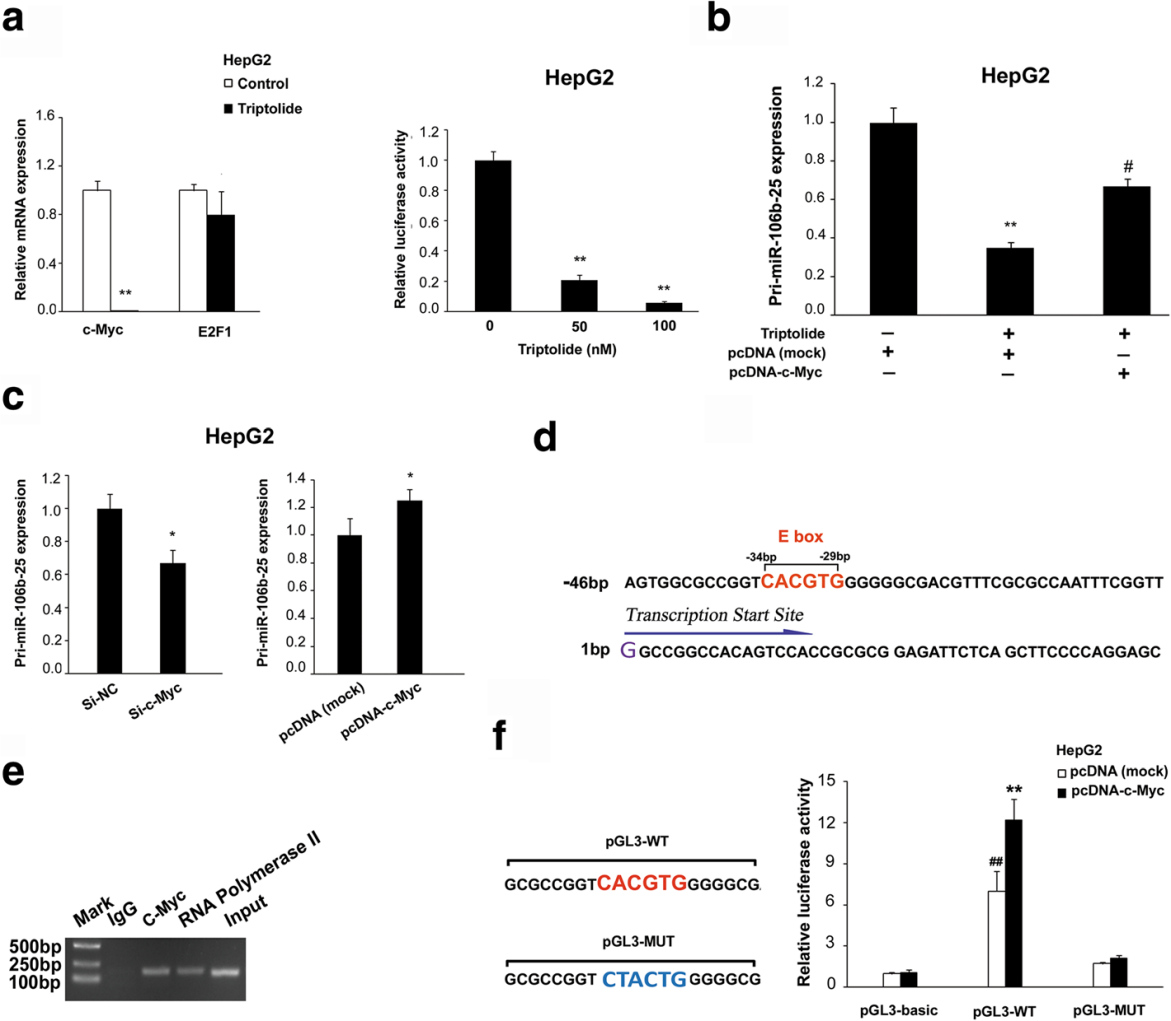
Triptolide down-regulated the expression of miR-17-92 and miR-106b-25 in a c-Myc-dependent manner. **a** Treatment of triptolide showed a significant decrease in the transcription of c-Myc, but the transcription of E2F1 was not significantly altered (left panel). Triptolide significantly repressed the transcriptional activity of c-Myc (right panel). pMyc-TA-luc and pRL-TK plasmids were cotransfected into HepG2 cells. **b** The forced expression of c-Myc counteracted the down-regulation of pri-miR-106b-25 induced by triptolide. HepG2 cells were transiently transfected with pcDNA-c-Myc or pcDNA (mock). After incubation for 48 h, transfected cells were treated with 100 nM triptolide for 24 h. Total RNA were extracted and subjected to qRT-PCR analysis (right panel). **c** Knockdown of c-Myc using si-RNA induced down-regulation of pri-miR-106b-25 (left panel). Conversely, overexpression of c-Myc up-regulated the pri-miR-106b-25 (right panel). **d** The schematics diagram of the genomic structure and localization of putative c-Myc binding sites in the promoter region of MCM7. **e** Chromatin immunoprecipitation assays demonstrated the enrichment of c-Myc s in 5′ flanking region of MCM7 containing putative binding sites. **f** A schematic of the nucleotide sequences of wild-type and mutant c-Myc binding sites in the promoter region of MCM7 (left panel) Luciferase reporter assays were performed to evaluate the transactivation potential of c-Myc binding sites containing sequences cloned from promoter region of MCM7 (right panel)

We further investigated the mechanism by which triptolide down-regulated the transcriptional level of miR-17-92 and miR-106b-25. It is well established that miR-17-92 is induced by c-Myc. We demonstrated that triptolide significantly reduced the protein level, mRNA level and transcriptional activity of c-Myc (Fig. 1d and Fig. 4a). Additionally, the overexpression of c-Myc with a mammalian expression vector pcDNA-c-Myc antagonized triptolide-induced inhibition of miR-17-92 (Additional file 6: Figure S4), thus demonstrating that triptolide reduced the expression of miR-17-92 through targeting c-Myc.

Then, we were interested in how triptolide reduced the expression of miR-106b-25. MiR-106b-25 is located in the intron 13 of the host gene MCM7, where it is cotranscribed in the context of MCM7 primary transcript. It has been previously reported that E2F1 activated the expression of both MCM7 and miR-106b-25. However, triptolide caused no significant alterations in the mRNA and protein levels of E2F1 (Fig. 4a and Additional file 3: Figure S1C), suggesting that E2F1 be not involved in the down-regulation of miR-106b-25 by triptolide. MYCN also promoted the expression of this miRNA cluster in specific cellular contexts [21]. However, the expression of MYCN has been found only in a restricted set of tumors, and the expression level of MYCN was quite low in HCC cells [22, 23]. Thus, MYCN is not associated with the triptolide-induced down-regulation of miR-106b-25 in these cells.

The Myc oncogene family members, c-Myc, MYCN, and MYCL, are basic-helix–loop–helix-leucine zipper domain containing transcription factors, which activate the transcription of a number of genes through binding to E-box element (CANNTG). It has been observed that MYCN and c-Myc can functionally replace each other and control the same cellular processes, suggesting that MYCN and c-Myc probably have very similar molecular functions [24]. Therefore, we speculated that c-Myc could also induce the transcription of miR-106b-25, and triptolide inhibit the transcription of these miRNAs through targeting c-Myc. We first investigated the correlation between miR-106b-25 and c-Myc. As shown in Fig. 4c, the transcriptional levels of miR-106b-25 were increased or decreased in response to the overexpression or knockdown of c-Myc, respectively. Furthermore, the overexpression of c-Myc antagonizes triptolide-induced inhibition of miR-106b-25, suggesting that triptolide-regulated expression of miR-106b-25 is also c-Myc-dependent.

We further investigated how c-Myc regulated the expression of miR-106b-25. Firstly, as shown in Fig. 4d, we identified one consensus E-box element (CACGTG) in the promoter region of MCM7 using the Eukaryotic Promoter Database (EPD, http://epd.vital-it.ch/). Then, we performed chromatin immunoprecipitation assays and confirmed that c-Myc directly binds to this E-box element (Fig. 4e). As shown in Fig. 4f, we cotransfected pcDNA-c-Myc with reporter plasmids pGL3-WT (containing wild type MCM7 promoter region) or pGL3-MUT (containing site-directed mutations in E-box element of MCM7 promoter region), and found that c-Myc overexpression significantly increased the luciferase intensity of pGL3-WT-transfected cells, thereby demonstrating that the interaction between c-Myc and this E-box element was required for the transcription of MCM7, in which the gene locus of miR-106b-25 located. Hence, c-Myc transactivated both miR-17-92 and miR-106b-25, and triptolide inhibited these two miRNA clusters through targeting c-Myc.

### Dysregulated c-Myc/miRNAs in HCC was associated with clinicopathologic factors

**Fig. 5:**
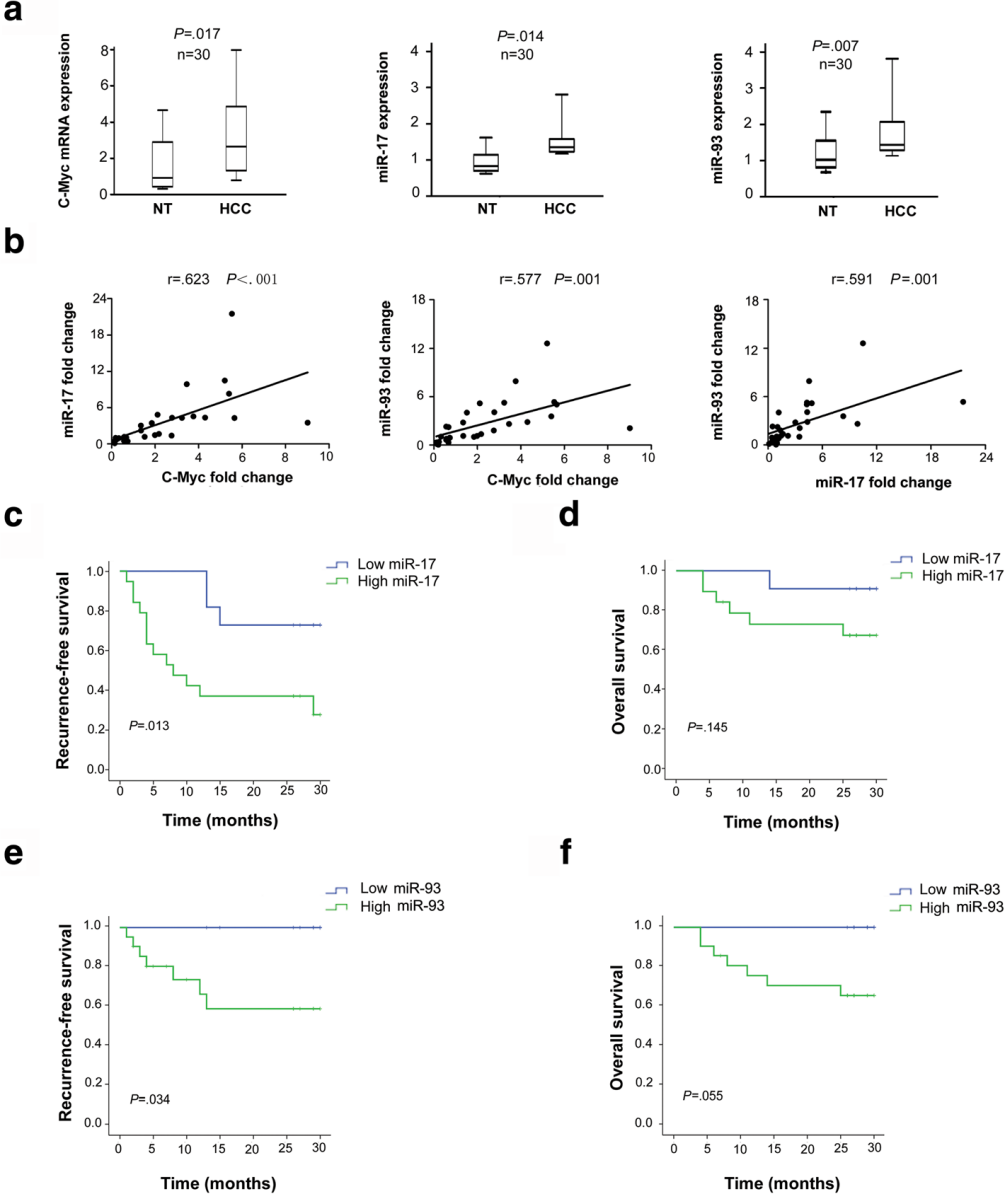
Up-regulation of miR-17 and miR-106b were associated with clinicopathologic factors. **a** c-Myc, miR-17 and miR-106b were significantly up-regulated in 30 human HCC tissues compared with the corresponding non-tumor tissues. The statistical differences were analyzed using the paired t-test. The horizontal lines in the box plots represented the median, the boxes represented the interquartile range, and the whiskers represented the 5th and 95th percentiles. **b** Correlation among the expression status of c-Myc, miR-17 and miR-106b. **c** and **d** Kaplan-Meier analyses of the correlations between the miR-17 expression level and the (**c**) recurrence-free survival or (**d**) overall survival of 30 HCC patients. **e-f** Kaplan-Meier analyses of the correlations between the miR-106b (**e**) recurrence-free survival or (**f**) overall survival of 30 HCC patients. The survival curves were compared using a Long-rank test

To further verify the correlation between c-Myc and these miRNAs, we quantified and compared levels of c-Myc, miR-17, and miR-93 between 30 HCC tissues and pared non-cancerous tissues using qRT-PCR (Fig. 5a and b). miR-17 and miR-93 were selected due to their relative high expression, the same seed sequence and targets and coordinate work. These data confirmed that the expression levels of all these genes were up-regulated and were positively correlated with each other in HCC. Interestingly, clinicopathologic analysis revealed that a higher miR-17 expression level was an independent indicator for a shorter recurrence survival (RFS) and a poorer differentiation status (Fig. 5c and Additional file 7: Table S3). However, the correlation between a higher miR-17 expression level and the overall survival (OS) was not statistically significant (Fig. 5d). Similar results were observed in high miR-93 expression group (Fig. 5e and f and Additional file 8: Table S4). Taken together, these data indicated that the high expression levels of miR-17 and miR-92 were consistently indicative of HCC patients’ recurrence.

### Triptolide inhibited the transcription of c-Myc through targeting ERCC3

**Fig. 6:**
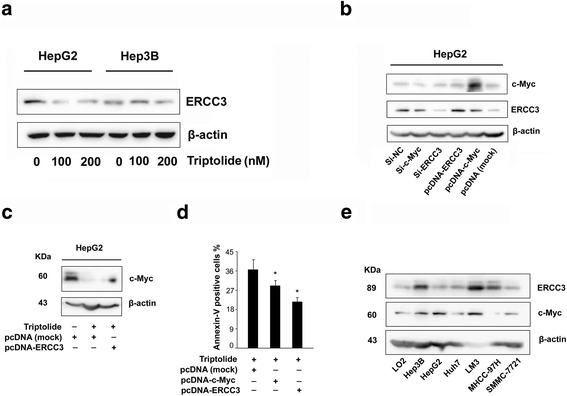
Triptolide suppressesed the transcription of c-Myc through targeting ERCC3. **a** Triptolide treatment showed a decrease in ERCC3 levels. **b** Relative mRNA (left panel) and protein expression (right panel) of ERCC3 and c-Myc in ERCC3/c-MYC-silenced and overexpressed HepG2 cells. **c** Over-expression of ERCC3 counteracted triptolide-induced c-Myc inhibition. **d** Over-expression of ERCC3 and c-Myc protected HepG2 cells from triptolide induced apoptosis. **e** The protein expression of ERCC3 and c-Myc was elevated in most of HCC cell lines tested

The primary targets of triptolide have been extensively explored in the past forty years. Several triptolide-binding proteins, including PC-2, ADAM10, DCTPP1, and ERCC3, have been identified as its potential primary targets [12]. Among those potential targets, ERCC3 seems to be essential. ERCC3 is a subunit of the general transcription factor TFIIH essential for the initiation of RNA polymerase II-dependent transcription. Meanwhile, ERCC3 is involved in nucleotide excision repair in response to DNA damage. Recently, Titov DV et al. [14] reported that triptolide covalently bound to ERCC3 and inhibited its DNA-dependent ATPase activity, which resulted in transcription inhibition. In this study, we also found that triptolide reduced the protein levels of ERCC3 (Fig. 6a). These findings drove us to explore whether triptolide inhibited c-Myc through targeting ERCC3. Using a combined overexpression and knockdown approach, we demonstrated that triptolide induced c-Myc inhibition in a ERCC3-dependent manner (Fig. 6b and c). And, expectedly, the overexpression of c-Myc and ERCC3 antagonized triptolide-induced apoptosis in HepG2 cells (Fig. 6d). More interestingly, the protein levels of ERCC3 were also increased or decreased in response to the overexpression or knockdown of c-Myc (Fig. 6b). However, c-Myc binding sites around the transcription start site (from− 3000 to + 500 bp) of ERCC3 still remained unknown. Thus, we speculated that c-Myc promoted the expression of ERCC3 in an indirect manner. Noticeably, the protein levels of c-Myc and ERCC3 were up-regulated in most of HCC cell lines compared with normal liver cell line LO2 (Fig. 6e), suggesting that inhibiting c-Myc/ERCC3 should be a feasible strategy for the treatment of HCC. In conclusion, triptolide inhibits the expression of c-Myc by targeting ERCC3.

## Discussion

Triptolide has been shown to be capable of killing a wide range of human cancer cells. However, the effect and mechanism of triptolide on HCC cells have not been well investigated. In this study, we showed that triptolide significantly inhibited proliferation of a couple of HCC cell lines with different p53 status (Fig. 1a). This inhibitory effect of triptolide could be attributed to the induction of apoptosis as evidenced by the activation of apoptotic markers (including PARP, caspase3, and caspase9) and pro-apoptotic signaling molecules (such as p53, p27, PTEN, and BIM),as well as the repression of some oncogenic genes, including Akt, Bcl-2, and c-Myc (Fig. 1b–d). It has been reported previously that triptolide induced cell-cycle arrest in several human malignancies, but our study did not show that triptolide exerted any effect on the cell cycle distribution of HCC cell lines (Additional file 3: Figure S1A and B). The in vivo efficacy of triptolide was also confirmed by xenograft study, in which triptolide significantly suppressed the growth of xenografted HepG2 tumor in nude mice (Fig. 1e).

Interestingly, apart from modulating the expression of numerous signaling molecules, triptolide was demonstrated to inhibit the expression of miR-17-92 and miR-106b-25, which resulted in the accumulation of their common target genes, including p21, PTEN, and BIM (Fig. 2c-f and Fig. 3d and e). Furthermore, our study provided compelling evidence that the inhibition of these two oncogenic miRNA clusters contributed to the antitumor activity of triptolide, because ectopic expression of these miRNAs protected HCC cells from triptolide-induced apoptosis (Fig. 3f).

Recently, a large number of studies have shown that miR-17-92 and its paralog miR-106b-25 played an important role in tumorigenesis [25]. These two miRNA clusters which interfered with cell cycle arrest and apoptosis when overexpressed in cancer cells are key modulators of TGFB signaling in a variety of human malignancies [26]. The overexpression of the miR-17–92 cluster has been observed in diverse tumor subtypes, such as those derived from breast, colon, lung, pancreas, prostate, and stomach [27]. Accordingly,the miR-106b-25 cluster was accumulated in different types of cancer, including pancreatic tumors, neuroblastoma, and multiple myeloma [6]. Interestingly, it was also reported that these two miRNA clusters were up-regulated simultaneously in several human cancers. Yet the underlying mechanism remains elusive [28, 29]. It is well established that miR-17-92 is directly transactivated by c-Myc. However, miR-106b-25 and its host gene MCM7 were cotranscribed induced by E2F1 or MYCN in different cellular contexts [21, 30]. In the current study, we further demonstrated that miR-106b-25 was induced by c-Myc in HCC cells. The c-Myc-dependent transactivation of miR-106b-25 was also direct because of the followings: (1) the transcriptional levels of miR-106b-25 were decreased or increased in response to the knockdown or overexpression of c-Myc, respectively (Fig. 4c); (2) chromatin immunoprecipitation experiments showed that c-Myc directly bound to specific sites located in the promoter region of MCM7, the host gene of miR-106b-25 (Fig. 4e); (3) reporter gene and point mutation experiments demonstrated that these binding sites were required for the transcription of miR-106b-25 (Fig. 4f); (4) the expression of miR-106b-25 was positively correlated with c-Myc in human HCC samples (Fig. 5b).

The c-Myc protein controls many cellular processes, including proliferation, cell cycle progression, cell growth, and angiogenesis [31]. In addition to modulating protein-coding genes, c-Myc also governed the expression of miRNAs. Elevated expression of c-Myc was one of the most common abnormalities in human malignancies [32]. Furthermore, these tumors are highly dependent on sustained c-Myc expression, while suppression of c-Myc could trigger senescence or cell death [33]. Moreover, it has been reported that targeting c-Myc-regulated miRNAs and their target genes could also be therapeutically feasible [34, 35]. Therefore, c-MYC/miRNAs/target genes axis has emerged as an attractive target for cancer therapy. Indeed, our study demonstrated that tripolide significantly inhibited the expression of c-Myc, which resulted in the suppression of miR-17-92 and miR-106b-25 and subsequent induction of apoptosis in HCC cells (Fig. 3d-f and Fig. 4a).

We further demonstrated that triptolide induced the inhibition of c-Myc through interfering with the activity of ERCC3, a well identified primary target of triptolide. Consistent with previous findings showing that ERCC3 was required for the transcription of c-Myc [3], the knockdown of ERCC3 lead to a decrease of c-Myc. Conversely, the overexpression of ERCC3 increased c-Myc expression and counteracted the triptolide-induced inhibition of c-Myc. More interestingly, their protein levels were increased or decreased in response to the overexpression or knockdown of each other, suggesting that a positive feedback loop exist between them (Fig. 6b). Given the importance of c-Myc and ERCC3 in gene transcription, this relationship merits further investigation.

## Conclusions

**Fig. 7:**
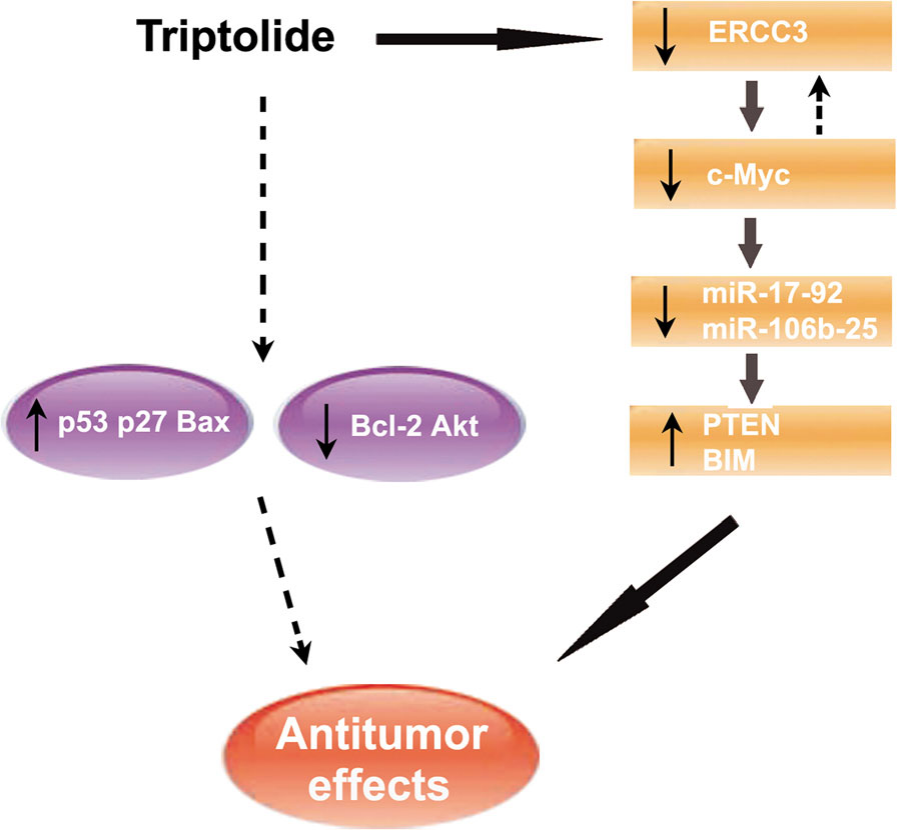
Schematic model of the proposed mechanisms for the antitumor activity of triptolide in HCC cells. Besides regulating a couple of signaling molecules, such as p53, Bcl-2, and Akt, triptolide inhibited the expression of miR-17-92 and miR-106b-25 through repressing c-Myc, resulting in up-regulation of their common target genes and subsequent cell death in HCC cells

In conclusion, besides regulating the expression of numerous signaling molecules, triptolide modulated the c-Myc/miRNA clusters/target genes axis through targeting ERCC3, which substantially enhanced its antitumor activities (Fig. 7). The capability of triptolide to induce cell death both in vitro and in vivo in several HCC cell lines makes it a potential therapeutic agent for HCC.

## Additional files

Additional file 1: **Table S2.** Oligonucleotide sequences used in this study. (DOC 44 kb)

Additional file 2: **Table S1.** Summary of clinicopathologic features. (DOC 49 kb)

Additional file 3: **Figure S1.** Triptolide did not alter the cell cycle distribution of HCC cells. **a**,**b** HepG2 and Hep3B cells were treated with indicated concentrations of triptolide for 12 hours. and Hep3B cells were treated with indicated concentrations of triptolide for 12 hours. Cell cycle analysis was performed using flow cytometry. **c** Protein levels of several cell cycle regulators were measured by western-blot. (TIFF 2606 kb)

Additional file 4: **Figure S2.** Triptolide did not cause obvious liver or kidney damages in nude mice. **a**,**b** The concentrations of serum alanine aminotransferase (ALT) and aspartate aminotransferase (AST), two common indicators of liver function, were measured by colorimetric analysis. **c**,**d** The concentrations of blood urea nitrogen (BUN) and creatinine (Creat), two common indicators of kidney function, were measured by colorimetric analysis. (TIFF 755 kb)

Additional file 5: **Figure S3.** Triptolide reduced pri-miR-17-92 and pri-miR-106b-25 expression in vivo. Xenografted tumors were obtained from nude mice treated with DMSO and triptolide, respectively (*n* = 3), and total RNA was extracted using Trizol reagent. The expression of pri-miR-17-92 and pri-miR-106b-25 was quantified by qRT-PCR. (TIFF 736 kb)

Additional file 6: **Figure S4.** The overexpression of c-Myc antagonizes triptolide-induced apoptosis in HepG2 cells. Cells were transfected with pcDNA-Mock or pcDNA-c-Myc then treated with triptolide. Cell apoptosis was measured using Annexin-V/PI double staining. (TIFF 735 kb)

Additional file 7: **Table S3.** Clinicopathologic characteristics of HCC subtypes defined by miR-17 expression. (DOC 46 kb)

Additional file 8: **Table S4.** Clinicopathologic characteristics of HCC subtypes defined by miR-93 expression. (DOC 47 kb)

## Abbreviations

DMSO: Dimethyl sulfoxide
HCC: Hepatocellular carcinoma
miRNA: microRNA
mRNA: messenger RNA
OS: Overall survival
PARP: Poly ADP ribose polymerase
PI: Propidium iodide
PTEN: Phosphatase and tensin homolog
qRT-PCR: quantitative reverse-transcription polymerase chain reaction
RFS: Recurrence free survival
