# Supplementary material for: C-Myc-dependent repression of two oncogenic miRNA clusters contributes to triptolide-induced cell death in hepatocellular carcinoma cells
Source: J Exp Clin Cancer Res. 2018 Mar 9;37:51. doi: 10.1186/s13046-018-0698-2 (PMC5845216; doi:10.1186/s13046-018-0698-2)
Supplement: Supplementary file 1 — Table S2. Oligonucleotide sequences used in this study. (DOC 44 kb) [file 13046_2018_698_MOESM1_ESM.doc]

**Table S2.** Oligonucleotide sequences used in this study

| Gene | Forward primer | Reverse primer |
| --- | --- | --- |
| pri-mir-17-92 | CAGTAAAGGTAAGGAGAGCTCAATC | CATACAACCACTAAGCTAAAGAATAATCT |
| pri-mir-106b-25 | TGTGGGTACTTGCTGCTCC | CTCAGCAGTAGGTTGGGTAATC |
| MCM7 | CCTGGACGTTTACATTGAGCA | CGCACTTCCCGGATCACAC |
| U6 | GCTTCGGCAGCACATATACTAAAAT | CGCTTCACGAATTTGCGTGTCAT |
| MYC | GAGGAGGAACAAGAAGATGAGG | ACCAGTGGGCTGTGAGGAG |
| E2F1 | TTGACCCAGGACCTCCGACAG | GGCTGATCCCACCTACGGTCT |
| ERCC3 | TGAACCCCAACAAATTTAGAGC | TGGCATATTCCTTTAGGGCAA |
| P21 | TGTCACTGTCTTGTACCCTTGT | GGTAGAAATCTGTCATGCTGGT |
| P27 | AACCGACGATTCTTCTACTCAA | TCCACAGAACCGGCATTT |
| P53 | GGCCATCTACAAGCAGTCAC | CACGCAAATTTCCTTCCACTCG |
| PTEN | ACCGCCAAATTTAATTGCAG | AAGTTGAACTGCTAGCCTC |
| BIM | AATAATTACCAAGCAGCCGAAG | CGGACAATGTAACGTAACAGT |
| β-actin | GGCACCACCATGTACCCTG | CCGGACTCGTCATACTCCT |
| si-RNA against MYC | GCCACAGCAUACAUCCUGUTT | ACAGGAUGUAUGCUGUGGCTT |
| si-RNA against ERCC3 | GCACCAAAGUGGAUGAAUATT | UAUUCAUCCACUUUGGUGCTT |
| pcDNA-c-Myc | CCCAAGCTTATGGATTTTTTTCGGGTAGTG | ACTCTAGATTACGCACAAGAGTTCCGTAG |
| pcDNA-ERCC3 | CCCAAGCTTATGGGCAAAAGAGACCGAG | GCTCTAGATCATTTCCTAAAGCGCTTG |
| pcDNA-miR17-92 | CCCAAGCTTCTAAATGGACCTCATATCTTTGAG | GCTCTAGAGAAAACAAGACAAGATGTATTTACAC |
| pcDNA-miR-106b-25a | CGCAAGCTTGGATCCTATCCTGCGCCTTTCC | TAATCTAGACACATGGCCACAGAAGAC |
| ChIP-PCR primers | TTGTCCGATTGGCCCGCACGCAG | AGCGCGTAGTCCTTCAGTGCCAT |
| Point mutagenesis | CGCAGTGGCGCCGGTCTACTGGGGGGCGACGTTTCG | GCGTCACCGCGGCCAGATGACCCCCCGCTGCAAAGC |
