# Supplementary material for: C-Myc-dependent repression of two oncogenic miRNA clusters contributes to triptolide-induced cell death in hepatocellular carcinoma cells
Source: J Exp Clin Cancer Res. 2018 Mar 9;37:51. doi: 10.1186/s13046-018-0698-2 (PMC5845216; doi:10.1186/s13046-018-0698-2)
Supplement: Supplementary file 2 — Table S1. Summary of clinicopathologic features. (DOC 49 kb) [file 13046_2018_698_MOESM2_ESM.doc]

**Table S1.** Summary of Clinicopathologic Features

| Characteristic | No. of patients |
| --- | --- |
| Patients | 30 |
| Gender |  |
| Male | 24 |
| Female | 6 |
| Age (y) | 35–71 (median, 47) |
| HBsAg |  |
| Positive | 30 |
| Negative | 0 |
| HBeAg |  |
| Positive | 10 |
| Negative | 20 |
| AFP |  |
| Positive | 23 |
| Negative | 7 |
| Tumor size (cm) | 2.0–16.1 (median, 4.8) |
| Cirrhosis |  |
| Yes | 24 |
| No | 6 |
| Microvascular Invasion |  |
| Yes | 5 |
| No | 25 |
| TNM stage |  |
| I | 12 |
| II | 11 |
| III | 7 |
| IV | 0 |
| Recurrence |  |
| Yes | 15 |
| No | 15 |
| Differentiation |  |
| Well differentiated | 18 |
| Poorly differentiated | 12 |
| Relapse-Free Survival | 2– 30 (median, 20.5) |
| Overall Survival | 4– 30 (median, 27) |

HBsAg, hepatitis B surface antigen; HBeAg, Heptitis B env*elope* antigen*,* AFP, serum alpha fetoprotein.
