# Supplementary figures and images for: C-Myc-dependent repression of two oncogenic miRNA clusters contributes to triptolide-induced cell death in hepatocellular carcinoma cells
Source: J Exp Clin Cancer Res. 2018 Mar 9;37:51. doi: 10.1186/s13046-018-0698-2 (PMC5845216; doi:10.1186/s13046-018-0698-2)

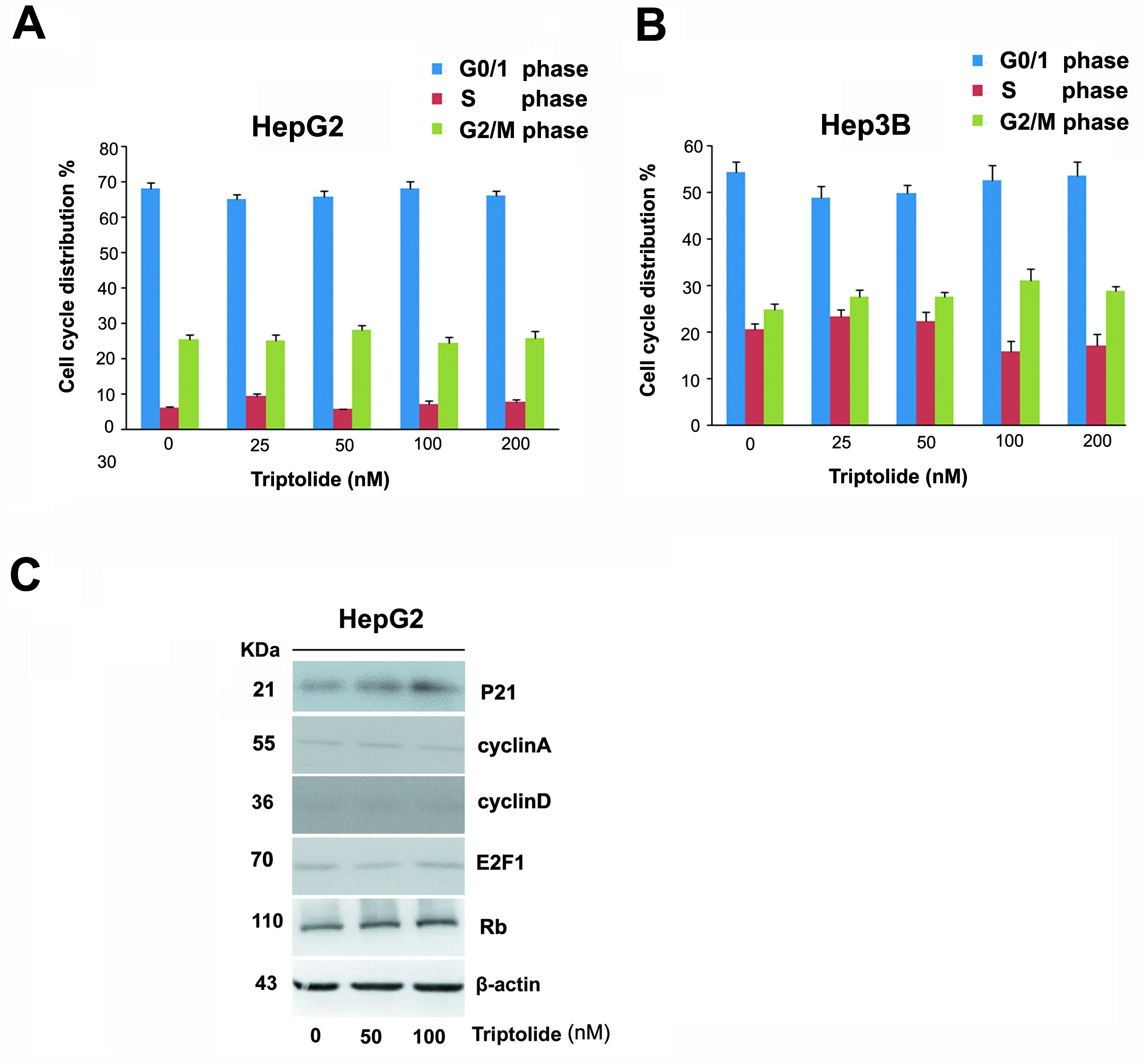

Supplement: Supplementary file 3 — Figure S1. Triptolide did not alter the cell cycle distribution of HCC cells. a,b HepG2 and Hep3B cells were treated with indicated concentrations of triptolide for 12 hours. and Hep3B cells were treated with indicated concentrations of triptolide for 12 hours. Cell cycle analysis was performed using flow cytometry. c Protein levels of several cell cycle regulators were measured by western-blot. (TIFF 2606 kb) [file 13046_2018_698_MOESM3_ESM.tif]

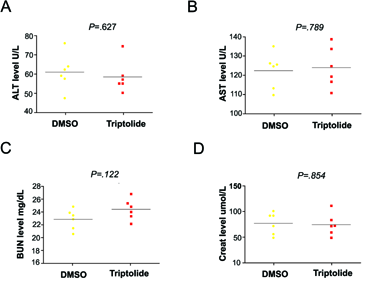

Supplement: Supplementary file 4 — Figure S2. Triptolide did not cause obvious liver or kidney damages in nude mice. a,b The concentrations of serum alanine aminotransferase (ALT) and aspartate aminotransferase (AST), two common indicators of liver function, were measured by colorimetric analysis. c,d The concentrations of blood urea nitrogen (BUN) and creatinine (Creat), two common indicators of kidney function, were measured by colorimetric analysis. (TIFF 755 kb) [file 13046_2018_698_MOESM4_ESM.tif]

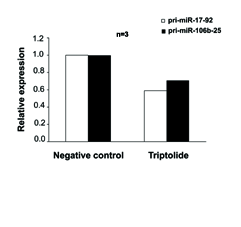

Supplement: Supplementary file 5 — Figure S3. Triptolide reduced pri-miR-17-92 and pri-miR-106b-25 expression in vivo. Xenografted tumors were obtained from nude mice treated with DMSO and triptolide, respectively (n = 3), and total RNA was extracted using Trizol reagent. The expression of pri-miR-17-92 and pri-miR-106b-25 was quantified by qRT-PCR. (TIFF 736 kb) [file 13046_2018_698_MOESM5_ESM.tif]

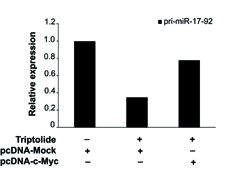

Supplement: Supplementary file 6 — Figure S4. The overexpression of c-Myc antagonizes triptolide-induced apoptosis in HepG2 cells. Cells were transfected with pcDNA-Mock or pcDNA-c-Myc then treated with triptolide. Cell apoptosis was measured using Annexin-V/PI double staining. (TIFF 735 kb) [file 13046_2018_698_MOESM6_ESM.tif]
