# Supplementary material for: C-Myc-dependent repression of two oncogenic miRNA clusters contributes to triptolide-induced cell death in hepatocellular carcinoma cells
Source: J Exp Clin Cancer Res. 2018 Mar 9;37:51. doi: 10.1186/s13046-018-0698-2 (PMC5845216; doi:10.1186/s13046-018-0698-2)
Supplement: Supplementary file 7 — Table S3. Clinicopathologic characteristics of HCC subtypes defined by miR-17 expression. (DOC 46 kb) [file 13046_2018_698_MOESM7_ESM.doc]

***Table S3.*** Clinicopathologic Characteristics of HCC Subtypes Defined by miR-17 Expression

| Variable | miR-17 expression | | *P* value ^a^ |
| --- | --- | --- | --- |
|  | High | Low |  |
| Gender |  |  | .567 |
| Male | 18 | 6 |  |
| Female | 5 | 1 |  |
| HBsAg |  |  |  |
| Positive | 23 | 7 |  |
| Negative | 0 | 0 |  |
| HBeAg |  |  |  |
| Positive | 8 | 2 |  |
| Negative | 15 | 5 | .571 |
| AFP |  |  |  |
| Positive | 18 | 5 |  |
| Negative | 5 | 2 | .532 |
| Cirrhosis |  |  |  |
| Yes | 17 | 7 |  |
| No | 6 | 0 | .170 |
| Microvascular Invasion |  |  |  |
| Yes | 5 | 0 |  |
| No | 18 | 7 | .236 |
| TNM stage |  |  |  |
| I+ II | 17 | 6 |  |
| III | 6 | 1 | .468 |
| Recurrence |  |  |  |
| Yes | 13 | 2 |  |
| No | 10 | 5 | .195 |
| Differentiation |  |  |  |
| Well differentiated | 11 | 7 |  |
| Poorly differentiated | 12 | 0 | .016 |

^a^ Statistical significance was calculated by chi-square test.
