# Supplementary material for: C-Myc-dependent repression of two oncogenic miRNA clusters contributes to triptolide-induced cell death in hepatocellular carcinoma cells
Source: J Exp Clin Cancer Res. 2018 Mar 9;37:51. doi: 10.1186/s13046-018-0698-2 (PMC5845216; doi:10.1186/s13046-018-0698-2)
Supplement: Supplementary file 8 — Table S4. Clinicopathologic characteristics of HCC subtypes defined by miR-93 expression. (DOC 47 kb) [file 13046_2018_698_MOESM8_ESM.doc]

***Supplementary Table 4.*** Clinicopathologic Characteristics of HCC Subtypes Defined by miR-93 Expression

| Variable | miR-93 expression | | *P* value ^a^ |
| --- | --- | --- | --- |
|  | High | Low |  |
| Gender |  |  | .600 |
| Male | 17 | 7 |  |
| Female | 4 | 2 |  |
| HBsAg |  |  |  |
| Positive | 21 | 9 |  |
| Negative | 0 | 0 |  |
| HBeAg |  |  |  |
| Positive | 5 | 5 |  |
| Negative | 16 | 4 | .104 |
| AFP |  |  |  |
| Positive | 16 | 7 |  |
| Negative | 5 | 2 | .657 |
| Cirrhosis |  |  |  |
| Yes | 17 | 7 |  |
| No | 4 | 2 | .600 |
| Microvascular Invasion |  |  |  |
| Yes | 4 | 1 |  |
| No | 17 | 8 | .521 |
| TNM stage |  |  |  |
| I+ II | 15 | 8 |  |
| III | 6 | 1 | .297 |
| Recurrence |  |  |  |
| Yes | 11 | 4 |  |
| No | 10 | 5 | .500 |
| Differentiation |  |  |  |
| Well differentiated | 10 | 8 |  |
| Poorly differentiated | 11 | 1 | .040 |

^a^ Statistical significance was calculated by chi-square test.
